# A Novel *Nodal* Enhancer Dependent on Pluripotency Factors and Smad2/3 Signaling Conditions a Regulatory Switch During Epiblast Maturation

**DOI:** 10.1371/journal.pbio.1001890

**Published:** 2014-06-24

**Authors:** Costis Papanayotou, Ataaillah Benhaddou, Anne Camus, Aitana Perea-Gomez, Alice Jouneau, Valérie Mezger, Francina Langa, Sascha Ott, Délara Sabéran-Djoneidi, Jérôme Collignon

**Affiliations:** 1 Institut Jacques Monod, UMR 7592, CNRS, Université Paris-Diderot, Sorbonne Paris Cité, Paris, France; 2 Unité de Biologie du Développement et de la reproduction, UMR INRA-ENVA, INRA, Jouy-en-Josas, France; 3 Epigenetics and Cell Fate, UMR7216, CNRS, Université Paris-Diderot, Sorbonne Paris Cité, Paris, France; 4 Centre d'Ingénierie Génétique Murine, Institut Pasteur, Paris, France; 5 Warwick Systems Biology Centre, University of Warwick, Coventry, United Kingdom; Osaka University, Japan

## Abstract

During early development, modulations in the expression of Nodal, a TGFβ family member, determine the specification of embryonic and extra-embryonic cell identities. *Nodal* has been extensively studied in the mouse, but aspects of its early expression remain unaccounted for. We identified a conserved hotspot for the binding of pluripotency factors at the *Nodal* locus and called this sequence “highly bound element” (HBE). Luciferase-based assays, the analysis of fluorescent HBE reporter transgenes, and a conditional mutation of HBE allowed us to establish that HBE behaves as an enhancer, is activated ahead of other *Nodal* enhancers in the epiblast, and is essential to *Nodal* expression in embryonic stem cells (ESCs) and in the mouse embryo. We also showed that HBE enhancer activity is critically dependent on its interaction with the pluripotency factor Oct4 and on Activin/Nodal signaling. Use of an in vitro model of epiblast maturation, relying on the differentiation of ESCs into epiblast stem cells (EpiSCs), revealed that this process entails a shift in the regulation of *Nodal* expression from an HBE-driven phase to an ASE-driven phase, ASE being another autoregulatory *Nodal* enhancer. Deletion of HBE in ESCs or in EpiSCs allowed us to show that HBE, although not necessary for *Nodal* expression in EpiSCs, is required in differentiating ESCs to activate the differentiation-promoting ASE and therefore controls this regulatory shift. Our findings clarify how early *Nodal* expression is regulated and suggest how this regulation can promote the specification of extra-embryonic precusors without inducing premature differentiation of epiblast cells. More generally, they open new perspectives on how pluripotency factors achieve their function.

## Abstract 2

HBE, a newly discovered enhancer element, mediates the influence of pluripotency factors and Activin/Nodal signaling on early Nodal expression in the mouse embryo, and controls the activation of later-acting Nodal enhancers.

## Author Summary

In the early mouse embryo, Nodal, a member of the TGFbeta superfamily of signalling proteins, promotes the differentiation of extra-embryonic tissues, as well as tissues within the developing embryo itself. Characterising the regulation of *Nodal* gene expression is essential to understand how Nodal signals in diverse tissue types and at different stages of embryonic development. Four distinct enhancer sequences have been shown to regulate *Nodal* expression, although none could account for it in the preimplantation epiblast or in embryonic stem cells. We identified a novel enhancer, HBE, responsible for the earliest aspects of *Nodal* expression. We show that activation of HBE depends on its interaction with a well-known pluripotency factor called Oct4. HBE itself also controls the activation of at least one other *Nodal* enhancer. Our findings clarify how early Nodal expression is regulated and reveal how pluripotency factors may control the onset of differentiation in embryonic tissues.

## Introduction

The gene *Nodal* encodes a TGFβ family member signaling via the Smad2/3-dependent Activin/Nodal pathway. Nodal is a key factor during early development, required for the specification of cell identities in embryonic and extra-embryonic lineages [1],[2]. Its re-expression in the adult has been associated with tumor progression and its signaling pathway is essential to the maintenance of human embryonic stem cells (ESCs) [3]–[5]. There is therefore a broad interest in understanding how its expression is initiated and regulated.

In the mouse, *Nodal* expression starts in the inner cell mass (ICM) of the E3.5 blastocyst [6],[7]. At E4.0, shortly before implantation, *Nodal* is detected in the two tissues that derive from the ICM: the epiblast, which will give rise to all fetal lineages, and the primitive endoderm (PrE), an extra-embryonic layer [6]. *Nodal* expression remains detectable in their postimplantation derivatives up to gastrulation stages but exhibits complex dynamics, foreshadowing the establishment of the anterior–posterior axis and the formation of the primitive streak [1]. Its re-expression in the node at E7.5 and in left lateral plate mesoderm at E8.0 contributes to the establishment of left–right asymmetry [1].

*Nodal* expression starts at E3.5, but the earliest molecular defects characterized in *Nodal* ^−*/*−^ embryos so far were detected after implantation. The epiblast of *Nodal* ^−/−^ embryos differentiates prematurely and their visceral endoderm, a derivative of the PrE, is not properly regionalized [8]–[10]. Pluripotent cell lines offer convenient *in vitro* models to study the role of *Nodal* and Activin/Nodal signaling during epiblast development. ESCs are derived from the nascent preimplantation epiblast [11]. They express *Nodal* and have an active Activin/Nodal signaling pathway, but this is not essential to their maintenance [3],[12]. In contrast, epiblast stem cells (EpiSCs) are derived from the postimplantation epiblast, and their capacity to self-renew depends critically on Activin/Nodal signaling [13],[14]. When exposed to Activin and FGF, ESCs can be converted into EpiSCs, a differentiation process dependent on Activin/Nodal signaling and described as a transition from a ground state of pluripotency to a primed state of pluripotency [11],[15]. This protocol is now commonly used to mimic events surrounding the maturation of the preimplantation epiblast into postimplantation epiblast.

Several studies showed that in ESCs *Nodal* expression is dependent on pluripotency factors or on Activin/Nodal signaling itself [16]–[19]. Four *Nodal* cis-regulatory elements are already known. None is controlled by pluripotency factors, and only one, ASE, is both dependent on Activin/Nodal signaling and known to be active before implantation [6],[20],[21]. ASE contains two functional FoxH1-Smad2,3 binding motifs and acts as an autoregulatory element allowing *Nodal* to amplify its own expression, notably in the postimplantation epiblast [20],[21]. The deletion of ASE results in a phenotype far less severe than that of *Nodal* ^−*/*−^ embryos and characterized by later patterning defects [20], indicating that it is not required to initiate *Nodal* expression. Our previous analysis of the expression profiles of fluorescent reporter transgenes for ASE showed that, although they could recapitulate some aspects of *Nodal* expression at preimplantation stages, they could not account for the timing of its onset in the ICM and its presence in nascent preimplantation epiblast cells [6]. This strongly suggested that these particular aspects of *Nodal* expression are dependent on cis-regulatory sequences other than ASE.

We sought to uncover how *Nodal* expression is initiated. We identified a novel *Nodal* enhancer, which we call HBE, that matches the expected profile. HBE is activated ahead of other *Nodal* enhancers in the ICM and in the preimplantation epiblast, and it is the predominant *Nodal* enhancer in ESCs. Furthermore, HBE is a hotspot for the binding of pluripotency factors and mediates the influence of Oct4, Klf4, and Activin/Nodal signaling on the expression of *Nodal*. The deletion of HBE by homologous recombination eliminates expression of the mutated allele in ESCs and in the early embryo. Strikingly, it also impairs its expression when ESCs are induced to differentiate, revealing an early requirement for HBE to trigger the activation of at least one other enhancer, the ASE, which drives *Nodal* expression in more differentiated cell types. We find also that the deletion of HBE in ESCs results in a region close to ASE accumulating the repressive histone mark H3K27me3, implying that it is via its implication in the recruitment of chromatin modifiers that HBE controls ASE. Our findings shed light on how enhancers regulated by the molecular machinery of pluripotency control gene expression and drive development forward.

## Results

### Identification of HBE, a Novel *Nodal* Enhancer Active in Pluripotent Stem Cells

**Figure 1:**
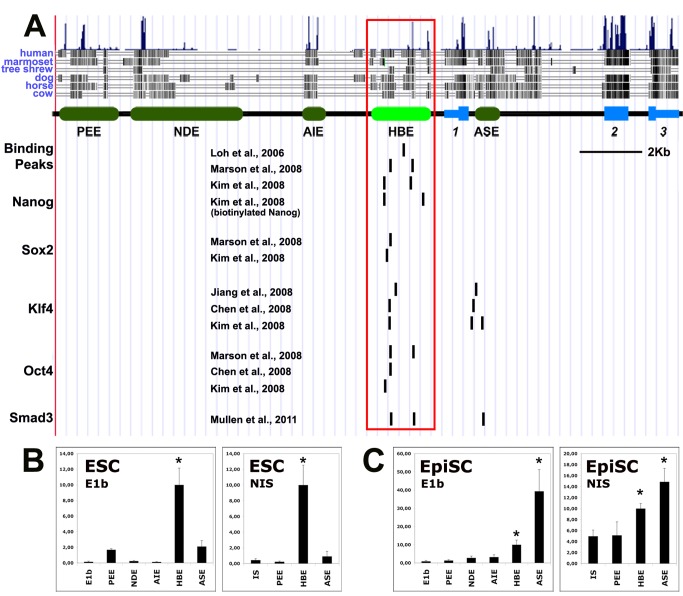
HBE is an enhancer active in pluripotent cells. (A) HBE is a hotspot for the binding of pluripotency factors and Smad3. *Nodal* regulatory elements are represented by green boxes and *Nodal* exons by blue boxes. Binding peaks of Nanog, Sox2, Klf4, Oct4, and Smad3 at the *Nodal* locus in ESCs are represented by black bars that represent either the summit of the peak of ChIP-seq data or its center for ChIP-chip data aligned to UCSC Genome Browser on Mouse Feb. 2006 (NCBI36/mm8) Assembly (http://genome.ucsc.edu/). (B and C) Luciferase reporter assays for early *Nodal* enhancers using either a minimal (E1b) or the endogenous promoter (NIS), in ESCs (B), or in EpiSCs (C). Luciferase activities are shown relative to HBE construct. An asterisk indicates significant differences from the control (ctrl) (*p*<0.01).

One study identified *Nodal* as a tentative direct target of the pluripotency factors Oct4, Sox2, and Nanog in ESCs [19]. It showed that the expression of *Nodal* declined when the gene encoding Oct4 was knocked down, whereas it was upregulated when *Nanog* or *Sox2* were supressed. We therefore searched relevant ChIP data, which revealed the existence of a hotspot for the binding of pluripotency factors, including Oct4, Nanog, Sox2, and Klf4, in a 2 kb region lying 1 kb upstream of the *Nodal* transcription start site (TSS) (Figure 1A) [22]–[26]. We called this region HBE, for highly bound element. This noncoding sequence is conserved in eutherian mammals, an indication that it may be involved in gene regulation (Figure 1A). In ESCs, this sequence scores positive for four criteria now used to identify active enhancers: low levels of the repressive histone mark H3K27me3, low levels of the active but promoter-associated histone mark H3K4me3, high levels of the active histone marks H3K4me1 and H3K27ac, and a binding peak of the acetyltransferase and transcriptional coactivator p300 [27]–[31] (Figure S1). In contrast, none of the known *Nodal* enhancers, PEE, NDE, AIE/LSE, or ASE [32]–[35], appeared to bear the hallmark of an active enhancer in ESCs (Figure S1). The ASE, however, although not bearing the active enhancer mark H3K4me1, presents marks suggestive of possible transcriptional activity: a binding peak for p300, high level of H3K27ac, and a peak of the active promoter-specific H3K4me3.

A luciferase-based assay was used to test HBE's capacity as an enhancer in ESCs and to compare it to that of ASE and PEE, the only *Nodal* enhancers known to be active at peri-implantation stages [6],[36]. This assay was done both with the minimal promoter E1b [36] and with the 940-bp-long stretch of sequence, termed NIS, for *Nodal* intervening sequence, which separates HBE from the ORF of the gene and contains the endogenous *Nodal* promoter. In both cases, HBE came out as the strongest enhancer (Figure 1B), whereas PEE and ASE showed minimal activity and NDE and AIE/LSE showed no activity whatsoever. We performed the same assay in EpiSCs. This time, although HBE still showed enhancer activity, the activity of ASE was higher while that of PEE, NDE, and AIE/LSE was unchanged (Figure 1C). The higher activity of ASE is consistent with it being dependent on Activin/Nodal signaling [6],[20],[34] and the presence of Activin in EpiSC culture medium. These results indicate that HBE is the predominant *Nodal* enhancer in ESCs and that it is still active in EpiSCs.

### An HBE Reporter Transgene Is Activated in Preimplantation Epiblast

**Figure 2:**
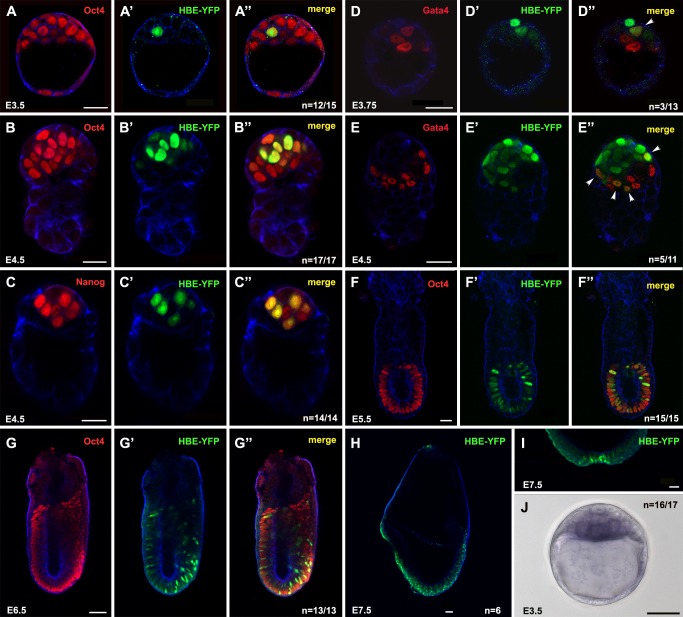
HBE-YFP expression is detected between E3.5 and E8.0. (A–A″, B–B″, F–F″, G–G″) Detection of Oct4 and HBE-YFP in E3.5 (A–A′), E4.5 (B–B′), E5.5 (F–F′), or E6.5 (G–G′) HBE-YFP transgenic mouse embryos. (C–C″) Detection of Nanog (C) and HBE-YFP (C′) in an E4.5 HBE-YFP transgenic mouse embryo (C″). (D–E″) Detection of Gata4 (D and E) and HBE-YFP (D′ and E′) in E3.75 (D″) or E4.5 (E″) embryos. Arrowheads indicate co-expressing nuclei. (H–I) Expression of HBE-YFP in the epiblast (H) and the node (I) of an E7.5 embryo. Images A to I are single confocal sections. Cortical actin in blue. *n* is the number of representative embryos on the total number of HBE-YFP embryos analyzed. (J) In situ hybridization for YFP in an E3.5 HBE-YFP embryo. *n* is the number of stained embryos on the total number of HBE-YFP embryos analyzed. Scale bar, 25 µm (except in G, H, I, and J where scale bar, 50 µm).

To find out when and where HBE is active during embryonic development, we generated transgenic lines where the expression of a nuclear version of Venus-YFP is placed under the control of HBE-NIS—that is, the 3 kb of genomic sequence directly upstream of the *Nodal* ORF. The two independent HBE-YFP mouse lines we obtained both showed the same reporter expression profile, thus precluding the influence of position and confirming its specificity (Figure 2). The fluorescence was first detected at E3.5 in one or two cells of the ICM (*n* = 12/15 embryos analyzed; Figure 2A–A″). By E4.5, more ICM cells were positive and the signal was stronger (Figure 2B–C″, 2E–E″). These cells all co-expressed the pluripotency factor Oct4 (Figure 2B–B″). Counts performed on E4.5 embryos stained for the PrE marker Gata-4 found that 93% of epiblast cells were YFP-positive. Most YFP-positive cells (98%) were also found to co-express the pluripotency factor Nanog (Figure 2C–C″). This is in marked contrast to the ASE-YFP transgene, which showed an expression profile broadly complementary to that of Nanog in the epiblast around the time of implantation [6], and suggests that HBE-YFP is expressed in epiblast cells earlier than ASE-YFP. However, at these early stages HBE-YFP expression is not restricted to the embryonic lineage. Co-expression with Gata-4 was detected in a subset of PrE cells in some embryos at E3.75 and E4.5 (*n* = 3/13 and *n* = 5/11, respectively; Figure 2D–D″, E–E″). There was no expression in extra-embryonic endoderm after this (unpublished data and Figure 2F–F″). After implantation, at E5.5, HBE-YFP was expressed in all epiblast cells, albeit with varying levels of intensity (*n* = 15/16; Figure 2F–F″). By E6.5, the expression of the transgene in the epiblast was clearly heterogeneous (*n* = 13/13; Figure 2G–G″), suggesting it was progressively downregulated in some cells whereas it was maintained in others. Between E6.5 and E7.5, HBE-YFP–positive cells could still be detected in the epiblast and in all epiblast derivatives, including the extraembryonic mesoderm (Figure 2G–G″ and unpublished data). However, they constituted a steadily declining fraction of these tissues. At E8.0, fluorescent nuclei were still detected in the node and in cells scattered in all three germ layers along the full length of the headfold stage embryo (Figure 2H,I). By E8.5, HBE-YFP expression was no longer detected (unpublished data).

Although HBE-YFP fluorescence just became detectable at E3.5, in situ hybridization with a YFP probe detected expression of the transgene in the ICM of all E3.5 transgenic embryos analyzed (*n* = 16/16; Figure 2J), whereas a similar analysis previously detected the ASE-YFP transgene in the ICM of no more than 50% of the embryos [6] (A.P.G. and J.C., unpublished data).

The expression profile of HBE-YFP does account for the early aspects of *Nodal* expression that were not fully recapitulated by the ASE transgene. It suggests HBE could be involved in the regulation of *Nodal* expression from its onset at E3.5 until late gastrulation stages.

### HBE Enhancer Activity Is Critically Dependent on a Single Oct4 Binding Site

**Figure 3:**
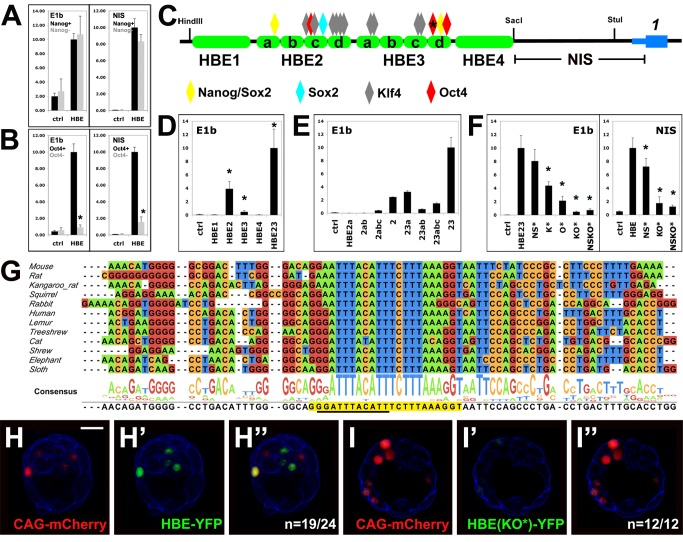
HBE enhancer activity depends on Oct4. (A, B, D–F) Luciferase reporter assays in ESCs using either a minimal (E1b) promoter or the endogenous sequence (NIS). (A) HBE activity before and after Nanog deletion in RCNβH cells. (B) HBE activity before and after Oct4 inactivation in ZHBTc4 cells. An asterisk denotes significant differences between Oct4+ and Oct4 – (*p*<0.01). (C) Positions of putative binding sites for Nanog, Sox2, Klf4, and Oct4 on HBE subregions 1 to 4 (Figure S3A). An asterisk indicates the main Oct4 binding site detailed in (G). (D) Transcriptional activity of HBE subregions. An asterisk indicates significant differences from control (ctrl) (*p*<0.01). (E) Transcriptional activity of HBE 2–3 serial deletions. (F) Effect of mutations on transcriptional activity of HBE 2–3. NS*, all Nanog and Sox2 sites are mutated; K*, all Klf4 sites are mutated; O*, main Oct4 site is mutated; NSKO*, all sites identified in (C) are mutated. An asterisk denotes significant differences from HBE 2–3 (*p*<0.01). (G) Conservation of the main Oct4 binding site in eutherian mammals. Canonical Oct4 binding site is underlined, and extended Oct4 binding is shaded in yellow. (H–I″) Electroporation of a WT (H) or a mutant (I) HBE-YFP construct in mouse blastocysts. mCherry was coelectroporated as a positive control. *n* is the number of YFP-positive (H) or negative (I) embryos on the total number of mCherry-positive embryos analyzed. Single confocal sections. Cortical actin in blue. Scale bar, 25 µm.

The fact that HBE is a hotspot for the binding of pluripotency factors in ESCs suggests that this sequence is the interface enabling these factors to modulate *Nodal* expression. To test this hypothesis we first assessed the influence of Oct4 and Nanog on HBE enhancer activity, using genetically modified ESC lines. RCNβH ESCs contain a conditional allele of Nanog, which can be deleted by exposure to Tamoxifen—triggering GFP expression [37]. Luciferase assays showed that the enhancer activity of HBE was not affected by the resulting absence of *Nanog* (Figure 3A), indicating that it is not via HBE that Nanog represses *Nodal* expression [19]. Successful deletion of *Nanog* was confirmed by the up-regulation of GFP and the downregulation of Nanog itself (Figure S2A–B″), whereas Oct4 expression was maintained (Figure S2C–D″). In contrast, in ZHBTc4 ESCs, where Doxycyclin treatment induces a knockdown of Oct4 [38], Oct4 depletion drastically down-regulated the expression of HBE constructs (Figure 3B), suggesting that HBE mediates the influence of Oct4 on *Nodal* expression [19]. Successful down-regulation of Oct4 was confirmed by immunofluorescence (Figure S2E–F′). However, these experiments could not establish whether the activity of HBE required a direct interaction between this enhancer and Oct4.

A systematic analysis was then undertaken to determine how the major pluripotency factors known to bind HBE contribute to its transcriptional activity in ESCs. Sequence comparison among eutherian mammals had uncovered four conserved regions within HBE, which we called HBE1 to 4 (Figure 3C). We used the BiFa bioinformatic tool [6],[39] to identify putative binding sites for Oct4, Nanog, Sox2, and Klf4 over the entire HBE sequences (Figure 3C and Figure S3A). Putative binding sites for Nanog/Sox2 (2), Sox2 (1), Klf4 (10), and Oct4 (3) were found in HBE2 and 3. Only these two regions showed significant enhancer activity, which was drastically increased when these two sequences were combined (Figure 3D). Fragments of HBE23 of increasing lengths were then assayed to identify subregions that are critical for this activity. Significant increases in enhancer activity were seen when fragments HBE2d, which contains a cluster of putative Klf4 binding sites, and HBE3d, which contains putative Oct4 and Nanog/Sox2 binding sites, were added to the reporter construct (Figure 3E). The addition of HBE3d resulted in the most dramatic gain in enhancer activity.

To assess the relevance of these binding sites to HBE enhancer activity, they were all mutated in HBE23-E1b and HBE-NIS luciferase constructs. Point mutations were designed with the help of the BiFa algorithm so as to prevent binding of the relevant transcription factor to its putative target sequence, while minimizing effects on the binding of other transcription factors. The impact of each mutation on transcription was first assessed separately and then in combination with others. We found that putative binding sites for all four factors—Nanog, Sox2, Oct4, and Klf4—were contributing to HBE23 enhancer activity in ESCs (Figure 3F). Mutations in Klf4 and Oct4 binding sites were, however, far more detrimental to this activity than mutations in Nanog and Sox2 binding sites. In particular, the elimination of the first Oct4 binding site in HBE3d was the single mutation causing the most dramatic drop in luciferase activity (Figure 3F). Its combination with mutations in the two other putative Oct4 binding sites did not reduce this activity further (Figure S3B). We confirmed that this single mutation was able to prevent the binding of Oct4 in gel shift assays with ESC extracts (Figure S4A).

Mutations in Nanog and Sox2 putative binding sites only had a significant impact on Luciferase activity when they were all combined in an NIS-driven construct, and still the decrease was modest (Figure 3F). The BiFa algorithm identified all putative Nanog binding sites in HBE as putative, lower ranking, Sox2 binding sites. In gel shift assays, extract from Nanog-depleted RCNβH ESCs slowed the migration of the target sequence we tested, indicating that it was bound by one other factor at least (Figure S4B). The mutated version of the sequence, however, prevented this binding, indicating that although some factor, such as Sox2, could possibly compensate for the absence of Nanog in RCNβH ESCs, our mutation allowed the contribution of their common binding sites to HBE and *Nodal* regulation to be assessed. Together with the Oct4 result, this suggested that our approach to mutation design was effective. We found that the addition of all Nanog and Sox2 mutated binding sites to a construct already containing all Klf4 and Oct4 mutated binding sites did not reduce its transcriptional activity further (NSKO*; Figure 3F), suggesting that the contribution of Nanog and Sox2 to HBE enhancer activity is secondary to that of Oct4 and Klf4.

Notably, we found that the first Oct4 binding site in HBE3d, the one most critical to HBE enhancer activity, is the most conserved of all the putative binding sites we identified in HBE, as it is the only one present in all mammalian genomes tested so far (Figure 3G). Furthermore, this conserved stretch of DNA contains an extended version of the Oct4 binding site that recent evidence suggests can be bound by Oct4 alone and is critical to its reprogramming function [40],[41].

To confirm the relevance of our findings to the regulation of HBE *in vivo*, we electroporated eight-cell stage embryos with constructs in which a nuclear version of Venus-YFP is under the control of either native HBE or its KO* version, where all Klf4 and Oct4 putative binding sites are mutated. Electroporation efficiency was assessed by co-electroporating a construct expressing mCherry under the control of the strong promoter CAG. Electroporated embryos were cultured 30 h, allowing most of them to reach the blastocyst stage. A majority of the embryos that had been electroporated with the native HBE construct (*n* = 19/24) showed YFP expression in a few cells. In contrast, embryos that had been electroporated with the mutated HBE-KO* construct showed only very weak or undetectable expression of YFP (12/12; Figure 3H–I″).

These results indicate that both in ESCs and in preimplantation embryos, HBE is under the control of pluripotency transcription factors, notably Oct4 and Klf4, whose cognate binding sites are critical to its enhancer activity.

### The Enhancer Activity of HBE Is Also Dependent on Activin/Nodal Signaling

**Figure 4:**
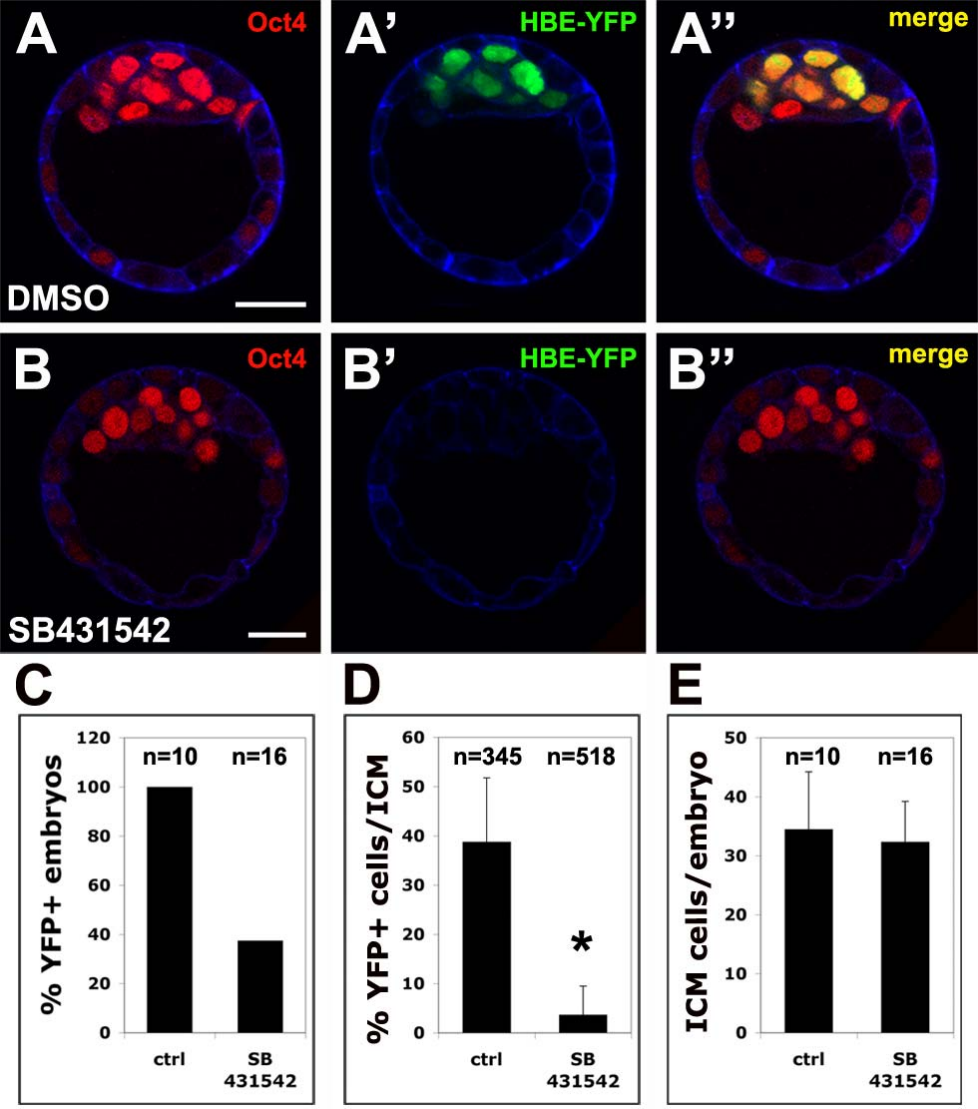
HBE-YFP expression in the blastocyst is dependent on Activin/Nodal signaling. (A–B) Detection of Oct4 (A, B) and HBE-YFP (A′, B′) in transgenic mouse blastocysts cultured either in DMSO (A–A″) or SB431542 (B–B″). Scale bar, 25 µm. Single confocal sections. Cortical actin in blue. (C) Percentage of YFP-positive embryos after 24 h culture in DMSO or in SB431542. (D) Percentage of YFP positive ICM nuclei in embryos after 24 h culture in DMSO or SB431542. An asterisk indicates significant difference from the control (ctrl) (*p*<0.01). (E) Number of Oct4-positive ICM cells per embryo after 24 h culture in DMSO or in SB431542.

The fact that not all E3.5 to E4.5 Oct4-positive ICM cells expressed HBE-YFP in transgenic embryos suggested that some other factor was essential for the activation of HBE. Several studies have shown that *Nodal* expression in ESCs is dependent on Activin/Nodal signaling [16]–[18]. Furthermore, a recent genome-wide ChIP study showed that, in ESCs, pSmad3 co-occupies the genome with Oct4, with which it forms a complex, and that this correlated with sensitivity to TGFβ signaling for Oct4-bound genes [42]. Notably, this study showed that with respect to *Nodal* expression, Oct-4 depletion led to a 5-fold reduction in its response to Activin exposure. Two of the positions where both Oct4 and Smad3 were found to bind are within HBE (Figure 1A). Our own results showed that reporter constructs and reporter transgenes for the Activin/Nodal signaling-dependent ASE had very limited transcriptional activity in ESCs (this study, and N. Sasaki, A.B., and J.C., unpublished results). Together, these data strongly suggested that Activin/Nodal signaling might be the other signal required to elicit HBE activation in preimplantation epiblast. To test this hypothesis, we cultured E2.5 HBE-YFP embryos for 48 h in the presence of 40 µM SB-431542, a pharmacological inhibitor of the type I Activin receptors ALK4, 5, and 7 [43]. We found that SB-431542–treated embryos had a similar number of Oct4-positive cells as DMSO-treated control embryos, indicating that at this concentration the formation of the ICM is not significantly affected (Figure 4A–E). SB-431542 exposure nevertheless resulted in a drastic reduction of the percentage of YFP-positive embryos and of YFP-positive cells among Oct4-positive ones. In addition, cells that expressed the transgene in SB-431542–treated embryos did so at a lower level than their counterparts in DMSO-treated embryos (unpublished data). We conclude that HBE-YFP expression is dependent on Activin/Nodal signaling, presumably reflecting a similar requirement for the activation of the endogenous HBE.

### HBE Conditions ASE Activation in Differentiating ESCs

**Figure 5:**
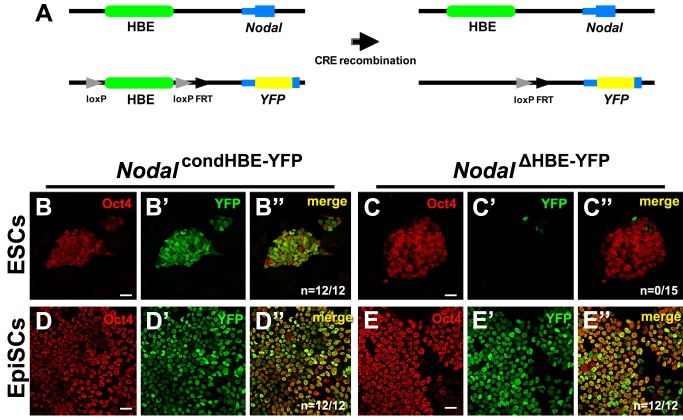
HBE is required for *Nodal* expression in ESCs but not in EpiSCs. (A) Depiction of the two *Nodal* alleles (WT on top and recombinant at the bottom) before and after Cre recombination. (B–C″) Expression of Oct4 (B, C) and YFP (B′, C′) in recombinant ESCs before (B–B″) and after (C–C″) Cre recombination. (D–E″) Expression of Oct4 (D, E) and YFP (D′, E′) in recombinant EpiSCs, 6 d after transfection with a control plasmid (D–D″) or with Cre recombinase (E–E″). Single confocal sections. *n* is the number of YFP-positive colonies. Scale bar, 25 µm.

Having established that HBE is an enhancer active in ESCs and in the mouse embryo, we assessed its contribution to *Nodal* expression. We generated a targeting construct in which HBE was floxed and the first 80 bp of *Nodal* ORF were replaced by the coding sequence for a destabilized nuclear Venus-YFP, so that the expression of the modified allele could be monitored (Figure S5A and Figure 5A). Successful targeting of the *Nodal* locus in ESCs was confirmed by PCR and Southern hybridization (Figure S5B–D). Almost all recombinant cells expressed the YFP, although at different levels (Figure 5B–B″). In contrast, Cre-mediated deletion of HBE resulted in most cells having completely lost YFP expression 2 d after transfection, indicating that HBE is essential to *Nodal* expression in ESCs (Figure 5C–C″). The few cells expressing YFP (∼7% of total) tended to be found at the periphery of colonies and to have low or no Oct4 expression, suggesting they corresponded to differentiating cells in which *Nodal* expression was driven by other enhancers.

To investigate this possibility, we analyzed the expression of the HBE-deleted allele in EpiSCs, where our luciferase-based assays had shown that ASE is the predominant *Nodal* enhancer. We thus induced ESCs carrying the conditional HBE allele *Nodal^condHBE-YFP^* to differentiate into EpiSCs. Real-time PCR (RT-PCR) analysis of the expression dynamics of four key markers—Klf4, Oct4, FgF5, and Bra—confirmed the successful conversion of the cells to an EpiSC identity (Figure S6A). RT-PCR analysis showed that the *Nodal^condHBE-YFP^* allele and the wild-type (WT) *Nodal* allele followed similar expression dynamics, indicating that the conditional allele is a fair reporter of WT *Nodal* expression (Figure S6B and unpublished data). EpiScs carrying the *Nodal^condHBE-YFP^* allele were then transfected with two constructs expressing either the Cre recombinase or the fluorescent marker mCherry. Widespread mCherry expression confirmed that transfection was efficient (Figure S6C–C′), whereas RT-PCR on genomic DNA showed that HBE deletion frequency was close to 90% 4 d after transfection (Figure S6D). We found that 6 d after transfection the expression of *Nodal^ΔHBE-YFP^* was maintained at a level similar to that of the undeleted allele (Figure 5D–E″, Figure S6E). This result indicates that HBE is not required for the expression of *Nodal* in EpiSCs, which is thus driven by another *Nodal* enhancer, presumably ASE.

**Figure 6:**
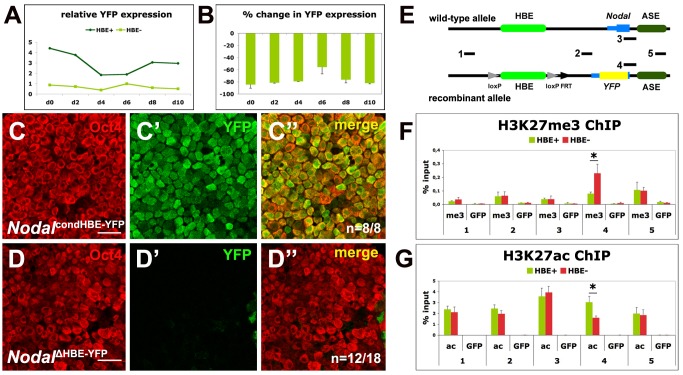
HBE is required to activate ASE during ESC to EpiSC differentiation. (A) RT-qPCR analysis of *YFP* expression during 10 d of ESC to EpiSC differentiation of *Nodal* ^condHBE-YFP^(HBE+) and *Nodal* ^ΔHBE-YFP^(HBE−) ESCs. One representative experiment. (B) Percentage of difference of *YFP* mRNA levels between *Nodal* ^ΔHBE-YFP^ and *Nodal* ^condHBE-YFP^ cells during 10 d of ESC to EpiSC differentiation. Error bars represent the mean + SD of triplicates and two independent experiments. (C–D″) Expression of Oct4 (C, D) and Venus-YFP (C′, D′) in *Nodal* ^condHBE-YFP^ (C–C″) and *Nodal* ^ΔHBE-YFP^ (D–D″) ESCs after 10 d of differentiation into EpiSC single confocal sections. *n* is the number of YFP-positive (C) or YFP-negative (D) samples on the total number of analyzed samples. Scale bar, 25 µm. (E) Part of the *Nodal* locus in the WT and the recombinant alleles comprising HBE, the first *Nodal* exon, and ASE and showing the position of regions 1–5 amplified in the ChIP experiments shown in (F) and (G). (F–G) ChIP with anti-H3K27me3 (F), anti-H3K27ac (G), or anti-GFP (F–G) antibodies on material from *Nodal* ^condHBE-YFP^ ESCs (green bars) and *Nodal* ^ΔHBE-YFP^ ESCs (red bars). The position in the locus of amplified regions 1–5 is shown in (E). An asterisk denotes significant differences between *Nodal* ^condHBE-YFP^ and *Nodal* ^ΔHBE-YFP^ ESCs (*p*<0.01).

To investigate the dynamics of the transition from an HBE-driven *Nodal* expression to an ASE-driven one, we induced ESCs carrying either the conditional HBE allele *Nodal^condHBE-YFP^* or the HBE-deleted allele *Nodal^ΔHBE-YFP^* to differentiate into EpiSCs. RT-PCR analysis showed, as expected, that the expression of *Nodal^ΔHBE-YFP^* was much lower than that of *Nodal^condHBE-YFP^* at the beginning (Figure 6A). Surprisingly, it did not recover, even after 10 d of differentiation. Comparison with the expression of the undeleted allele showed an average difference of about 80%, and immunofluorescence detected the YFP in just a few cells (Figure 6B–D″). Like in ESC colonies, these rare YFP-positive cells had lower or no Oct4 expression (Figure 6D″). Together with the earlier finding that HBE is not required for *Nodal^ΔHBE-YFP^* expression in EpiSCs, this indicates that prior to its deletion in EpiSCs, HBE contributed to a modification of the locus critical for the activation of ASE, which allowed *Nodal^ΔHBE-YFP^* to be expressed in EpiSCs. These results demonstrate that during the conversion of ESCs into EpiSCs, HBE is initially required to promote the activation of ASE.

As ASE is dependent on Activin/Nodal signaling and as Nodal in *Nodal^ΔHBE-YFP^* cells is still produced by the WT allele, we hypothesised that HBE is required to potentiate the activation of ASE at the chromatin level. We used ChIP to track changes in the distribution of the mutually exclusive H3K27me3 and H3K27ac histone marks at different positions in the locus. This analysis revealed that after HBE deletion, a region 5′ to the ASE sees a 2.5-fold increase of the repressive H3K27me3 mark and a 2-fold decrease of the active H3K27ac mark. These modifications are specific to the recombinant allele. No changes were detected at the 3′ end of the autoregulatory enhancer. No changes either were detected immediately upstream and downstream of the deleted HBE (Figure 6E–G). This result demonstrates that HBE controls the chromatin status of a region adjacent to ASE and therefore suggests that it is via the recruitement of chromatin modifiers that HBE exerts an influence over ASE activation.

### HBE Is Required for *Nodal* Expression in the Mouse Embryo

**Figure 7:**
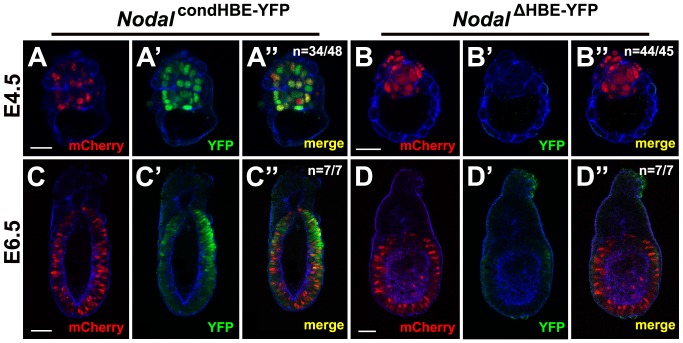
HBE is required for activation of *Nodal* in the early mouse embryo. Detection of mCherry (A, B, C, and D) and YFP (A′, B′, C′, and D′) in E4.5 (A–B″) or E6.5 (C–D″) aggregation chimeras generated from *Nodal^condHBE-YFP^* (A–A″ and C–C″) or *Nodal^ΔHBE-YFP^* (B–B″ and D–D″) ES cells and WT embryos. Images are single confocal sections. Cortical actin in blue. *n* is the number of representative embryos on the total number of embryos analyzed. Scale bar, 25 µm for E4.5 embryos and 50 µm for E6.5 embryos.

To investigate whether HBE is necessary for the expression of *Nodal* in vivo as it is in vitro, chimeric embryos were generated. *Nodal^condHBE-YFP^* and *Nodal^ΔHBE-YFP^* cells were first stably transfected with mCherry so that they could be traced in chimeric embryos. Small groups of these cells were then aggregated with E2.5 morulae, and the resulting blastocysts were either cultured in vitro until the equivalent of stage E4.5 or reimplanted into pseudopregnant mice and allowed to develop in utero until the equivalent of stage E6.5. Chimerism was very high as judged by the number of mCherry-positive cells in the epiblast of the aggregation chimeras. Embryos generated from *Nodal^condHBE-YFP^* cells expressed YFP in the epiblast (*n* = 34/48 of stage E4.5 and 7/7 of stage E6.5 embryos analyzed; Figure 7A and C), and this expression was consistent with the expected expression profile for *Nodal*, notably showing a restriction to the proximal posterior epiblast at E6.5. In contrast, embryos generated from *Nodal^ΔHBE-YFP^* cells did not express the fluorescent marker or expressed it at very low levels in just a few cells (*n* = 44/45 of stage E4.5 and 7/7 of stage E6.5 embryos analyzed; Figure 7B and D), indicating that HBE is required for the activation of *Nodal* transcription in epiblast cells in vivo, as in vitro differentiation experiments suggested.

## Discussion

### HBE Is an MTL at the *Nodal* Locus

Genome-wide ChIP studies have shown that in ESCs, pluripotency factors co-occupy the genome at specific multitranscription factor-binding loci (MTL) through which they control the pluripotent state of the cells [22],[24]–[26],[44]. These studies led to the view that the core transcription factors of the pluripotency gene regulatory network (GRN), Oct4, Nanog, and Sox2, form an interconnected autoregulatory loop that positively regulates their own promoters, activate the expression of genes necessary to maintain the pluripotent state, and contribute to the repression of genes promoting differentiation [45]–[47]. We identified HBE as an MTL at the *Nodal* locus. Our results confirm that this region is a target of the molecular machinery of pluripotency and of the Activin/Nodal signaling pathway, as ChIP studies predicted [22],[24]–[26],[42].

### HBE Enhancer Activity Depends on Pluripotency Factors and Activin/Nodal Signaling

We found that HBE has enhancer activity in ESCs, as was the case for all Oct4/Sox2/Nanog MTLs tested so far [22],[47]. HBE is in fact the only *Nodal* enhancer active in ESCs. Moreover, it is activated early on during mouse embryonic development. Transgenic embryos expressing YFP under the control of HBE up-regulate the fluorescent marker in the ICM of the E3.5 blastocyst. Its expression is then restricted to the embryonic epiblast and is maintained in its embryonic and extra-embryonic derivatives until organogenesis starts at E8.5, at which point *Oct4* expression and pluripotency are lost [48].

We showed that the enhancer activity of HBE is dependent on Oct4 and Klf family members. In fact Oct4 is the master pluripotency factor most critical to this activity. This is consistent with studies suggesting that unlike other master pluripotency factors, Oct4 is a strong transcriptional activator [49]. It appears to function as a pioneer factor at enhancers, opening up the chromatin and allowing other factors, such as pSmad3, to access their binding sites [42]. The main Oct4 binding site in HBE is the only one of all the putative pluripotency factors binding sites we identified that is extensively conserved among placental mammals, suggesting that HBE evolved around this particular sequence.

We also found that the enhancer activity of HBE is dependent on Activin/Nodal signaling and we showed previously that Activin/Nodal signaling is activated in *Nodal* ^−*/*−^ blastocysts [6]. In other animal models, there is consistent evidence of another TGFβ family member acting upstream of early *Nodal* expression [50]–[54]. Gdf1 and Gdf3, two possible TGFβ-related candidates in the mouse, appear however unable to activate the Smad2/3 pathway at physiological concentrations [55]–[57]. This was confirmed when we showed that *Gdf3* cannot replace *Nodal* in vivo [6]. Better candidate ligands for the early activation of the Smad2/3 pathway and of HBE are thus Activins, which are present in the ICM as well as in the oviduct and uterine epithelia prior to implantation [58]. Because *Nodal* was also found to be expressed in the endometrium of E3.5 pregnant females, one cannot discount the possibility that Nodal of maternal origin might be involved in the induction of *Nodal* expression in the embryo [59].

### *Nodal* Expression Undergoes a Regulatory Shift During Epiblast Maturation

The finding that the onset of *Nodal* expression is dependent on the pluripotency GRN coincides with a growing realization that in the context of the embryo so-called pluripotency factors are in fact actively engaged in promoting development. Nanog, described as the guardian of pluripotency in ESCs [37], is required in epiblast precursors to promote, by a non-cell-autonomous mechanism, the differentiation of adjacent PrE precursors [60]. It has also been shown recently that Oct4 promotes PrE development through both cell-autonomous and non-cell-autonomous mechanisms, and more generally favors embryo development via its control of multiple metabolic pathways [61]. Recent work indicates that Activin/Nodal signaling may first be required in the PrE around E4.0 to specify a subset of *Lefty1*-expressing PrE cells, the descendants of which will later give rise to the distal visceral endoderm (DVE), a group of cells playing a critical role during the establishment of AP polarity [2],[7]. It is therefore possible that the HBE-dependent expression of *Nodal* in the blastocyst contributes to this initial regionalization of the PrE. During the transition from pre-implantation to postimplantation epiblast, *Nodal* undergoes a regulatory shift, from an HBE-driven phase to an ASE-driven one, which correlates with an increase in its expression levels and an up-regulation of differentiation promoting downstream targets, also seen in EpiSCs [6],[13],[14]. In ESCs, most genes involved in lineage specification are in a poised state that is transcriptionaly silent but ready to be activated by developmental signals. This state is defined by the presence of both active and repressive histone marks on the promoters of these genes. Repressive marks are introduced by chromatin modifiers locally recruited by Oct4, Sox2, and Nanog [47]. Smad2/3 complexes, activated by the Activin/Nodal pathway, can remove these repressive marks and induce the expression of downstream targets such as *Gsc* and *Mixl1.* Yet although *Nodal* is expressed in ESCs, *Gsc* and *Mixl1* remain poised in these cells. This can be partly explained by the relatively low level of *Nodal* expression in ESCs and by the co-expression of genes known to restrain its signaling activity, such as *Smad7*, *Lefty1*, and *Lefty2*. These data suggest that in the blastocyst components of the Activin/Nodal signaling pathway are tightly regulated to ensure proper embryonic and extra-embryonic development. Initially, activation of *Nodal* by HBE produces low levels of the signal that specify certain extra-embryonic precusors, possibly of the DVE, while minimizing the exposure and the response of nascent epiblast to prevent its premature differentiation. During subsequent stages of development the autoregulatory ASE takes over. This shift from an HBE-driven phase to an ASE-driven one results in an amplification of the Nodal signal, which triggers the differentiation of the epiblast.

We found that HBE is required in differentiating ESCs for the activation of ASE. When HBE is deleted in EpiSCs, ASE, the predominant *Nodal* enhancer in this cell type, is active. However, if HBE deletion occurs in ESCs, before their differentiation into EpiSCs, ASE does not drive expression of the gene. Our results suggest that once bound to HBE, master pluripotency factors induce local modifications of the chromatin that in turn affect the ability of the ASE to interact with the adjacent promoter, and thus *Nodal* expression levels. Changes in the combination of HBE-bound factors, such as those taking place during epiblast maturation or ESC to EpiSC transition, could modify the effect HBE has on ASE.

### Nanog and Oct4 Are Possible Players in the HBE to ASE Transition

Although *Nodal* is expressed in ESCs, the autoregulatory enhancer ASE is not active in these cells. One hypothesis is that Nanog acts at the *Nodal* locus to prevent ASE activation. We found previously that the expression of the ASE-YFP reporter transgene is only detected in epiblast cells with low or no Nanog [6]. This is consistent with the results of luciferase assays in ESCs and EpiSCs that correlate a higher level of ASE transcriptional activity with a lower level of Nanog. Nanog depletion in ESCs results in an increase in *Nodal* expression [19], yet we found that Nanog depletion, or the elimination of Nanog binding sites, had no effect on the transcriptional activity of HBE. Because Nanog binds only HBE at the *Nodal* locus in ESCs, it must act from this position to prevent ASE activation. This would keep *Nodal* expression, and thus Activin/Nodal signaling, low as long as Nanog is present. Its down-regulation during the conversion of ESCs into EpiSCs signaling by unlocking ASE would then allow an increase in Activin/Nodal.

The dependency of ASE activity on HBE may also involve Oct4, but in a role opposite to that proposed for Nanog. HBE-bound Oct4 could promote ASE activation. The mechanism described for the activation of poised genes by companion Trim33-Smad2/3 and Smad4-Smad2/3 complexes [62] suggests a similar scenario for the HBE-dependent activation of ASE. The Oct4-Smad3 complex bound on HBE could initiate chromatin modifications that would then allow the interaction of ASE with the adjacent promoter, leading to the transcriptional activation of *Nodal* by the autoregulatory element and the amplification of the Nodal signal. The results obtained in aggregation chimeras suggest that ASE may not be the only *Nodal* enhancer whose activation is controlled by HBE. The lack of expression of the *Nodal^ΔHBE-YFP^* allele in proximal and posterior epiblast cells at E6.5, where *Nodal* expression was shown to be independent of ASE, but where transgenic PEE reporters were found to be expressed [6],[20],[63], do suggest a similar influence on PEE.

The implication of Oct4 in such an unlocking mechanism would be consistent with recent studies showing that the capacity of ESCs to differentiate is critically dependent on the level of Oct4 not being too low [64],. Such a mechanism may concern the regulation of differentiation-promoting genes other than *Nodal*. Further studies will be necessary to test these hypotheses and get a better understanding of how HBE-bound factors contribute to the regulation of *Nodal* expression.

**Figure 8:**
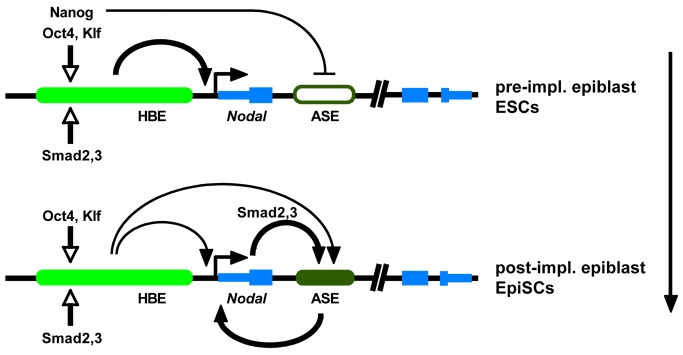
Model for regulatory shift from HBE to ASE during epiblast maturation. (A) In the late preimplantation epiblast and in ES cells, pluripotency factors (mainly Oct4) and Nodal/Activin signaling activate HBE, which up-regulates *Nodal.* However, Nanog bound on HBE represses ASE so that expression levels of *Nodal* remain low. (B) In the postimplantation epiblast and in EpiSCs, changes in the combination of HBE-bound factors allow ASE to take over from HBE as the predominant enhancer driving *Nodal* expression, and the positive regulatory loop between *Nodal* and ASE is established, leading to higher expression levels.

To conclude, our results complete the picture on the regulation of *Nodal* at early stages. They show that HBE has a dual role, acting both as an enhancer and as a modulator of the activity of other regulatory elements. Our analysis of its regulation and mode of action furthers our understanding of the distinct roles assumed by master pluripotency factors and of the complex fashion in which the molecular machinery of pluripotency controls gene expression (Figure 8). It is likely that similar mechanisms are involved in the regulation of genes other than *Nodal*. Our results are consistent with the notion that the need to control Activin/Nodal signaling is one of the leading influences on the evolution of the pluripotency GRN.

## Materials and Methods

### Ethics Statement

Experiments were performed in accordance with French Agricultural Ministry and European guidelines for the care and use of laboratory animals. The project has been reviewed and approved by the Animal Experimentation Ethical Committee Buffon (CEEA-40). It is recorded under the following reference: CEB-35-2012.

### Bio-Informatics Analysis

Potential binding sites at endogenous and mutated sequences were scored statistically using the Binding Factor (BiFa) tool [6]. Weight matrices from the TRANSFAC database v2009.4 [66] were used. The alignment of the main Oct4 binding site was retrieved from the Ensembl database release 73. It belongs to «36 eutherian mammals EPO LOW COVERAGE» (positions 61,416,797 to 61,416,833 on mouse chromosome 10). The alignment was visualized using Jalview 2.8 [67] using data for a subset of available species.

### ES Cell Culture and Transfection

See Materials and Methods S1 for detailed CCE, ZHbTc4, and RCNbH mouse ES cell culture conditions. Inhibition of Oct4 expression in ZHbTc4 cells was induced with 0.1 mg/ml Doxycyclin (Sigma), whereas Nanog knock-down in RCNbH cells was induced with 1 µM 4-Hydroxy-Tamoxifen (Sigma). We transiently transfected 200,000 ES cells with 1 µg of any of Firefly Luciferase constructs and 0.05 µg of the pCAG-Renilla Luciferase construct (in 50 µl DMEM) and 2 µl Lipofectamine 2000 (Invitrogen—in 50 µl DMEM) according to the manufacturer's instructions and harvested them 24 h after transfection.

### ESC to EpiSC Differentiation

ES cells were grown as previously described [13]. EpiSC-like colonies start to appear at passage 3 (day 6), and colonies were passaged by mechanical dissociation after 30 s treatment with accutase at room temperature. Colonies were passaged every 2 d and diluted 3 to 4 times.

### Site-Directed Mutagenesis

Site-directed mutagenesis of HBE was performed by two rounds of PCR amplification. First, complementary primers containing the point mutations as well as primers complementary to the 5′ or the 3′ ends of the sequence were used to amplify the two parts of HBE that contain the mutated sequence at one end. Then, the two parts were used as the template for the amplification of the whole sequence, using the end primers alone. Multiple point mutations were introduced sequentially. See Materials and Methods S1 for primer sequences.

### Luciferase Assay

The luciferase activities of the cell lysates were measured by means of the Dual-Luciferase Reporter Assay System (Promega) in a Berthold Centro LB 960 device. The activity of the firefly luciferase was measured for 60 s, whereas the activity of the Renilla luciferase was measured for 0.5 s. Finally, the normalised values for HBE and HBE23 were arbitrarily set to 10. Activities are reported as mean standard errors of a minimum of three independent experiments.

### RT-PCR

Total RNA was prepared using NucleoSpin RNA Kit (MN) followed by DNaseI (Roche) treatment. First-strand cDNA was synthesised using Vilo reverse transcriptase (Invitrogen). Real-time PCR was performed using FastStart SYBR Green Master (Roche). Gene expression was determined relative to Gapdh using standard curve calibration. All quantitative PCR reactions were performed in LightCycler 480 (Roche). See Materials and Methods S1 for primer sequences.

### Reporter Constructs and Transgenesis

A DNA construct expressing Venus-YFP fused to 3 NLS was linearised, gel-purified, and resuspended in Tris 10 mM, EDTA 0.25 mM, pH 7.5. Transgenic founders were obtained after microinjection of the DNA into (C57BL/6 × CBA) F2 fertilized eggs (1 or 2 ng/ml in injection buffer). Heterozygous embryos carrying the HBE-Venus transgene were generated by mating homozygous transgenic males with WT Sw females. The genotyping was done as described for the ASE-YFP transgene [6].

### Embryo Collection, Electroporation, and Culture

Mice mating and embryo collection were as described [6]. Eight-cell stage uncompacted Swiss × Swiss mouse embryos were collected in M2 (Sigma), shelled in Tyrode's solution (Sigma), and electroporated in a flat electrode chamber with a 1 mm gap between the electrodes (BTX Inc., San Diego, CA) in 1× HBS DNA solution containing 0.25 µg/µl of the mCherry expressing control plasmid and 1 µg/µl of the Venus expressing experimental plasmid. Two sets of four pulses of 1 ms each at 25 V were delivered, with 100 ms intervals between the pulses and a 1 min interval between the two sets of inverted polarity. The embryos were then cultured in G2 (Vitrolife) at 37°C and 5% CO_2_ for 30 h.

### Inhibition of ALK4/5/7 Receptors

Eight-cell stage uncompacted transgenic ASE-YFP embryos were transferred to an eight-well Netwell plate (Costar) with 400 µl of G2v5PLUS (Vitrolife). They were cultured for 48 h at 37 °C/5% CO_2_ in the presence of 20, 40, or 50 µM SB-431542 (Sigma) in DMSO, to test for dose toxicity and effectiveness. Control embryos were cultured in the presence of the same amount of DMSO. We found as previously that treatment with 40 µM SB-431542 was required to significantly decrease the activity of the ASE-YFP transgene [6]. This dose was not toxic for cultured embryos and was thus chosen to perform similar inhibition experiments on eight-cell stage uncompacted transgenic HBE-YFP embryos.

### Immunofluorescence

Cells on coverslips were fixed in 4% paraformaldehyde, permeabilized in PBS/0.3% Triton blocked with 10% FBS in PBS, and incubated with the primary and secondary antibodies (diluted in blocking solution). Nuclei were marked with DAPI- D9564 (Sigma) and cortical actin was marked with 0.5 µg/ml Alexa 647-conjugated Phalloidin (both Molecular probes) and the coverslips mounted on slides with Mowiol 4–88 (Sigma). Immunofluorescence on embryo were done as described [6]. See Materials and Methods S1 for antibody combinations.

### In Situ Hybridization

ISH was performed as described previously [6].

### Homologous Recombination

16×10^6^ CK35 ES cells were transfected with 20 µg of linearised homologous recombination construct containing 12 Kb of the *Nodal* locus with Venus-YFP fused to three NLS and a PEST sequence replacing the first exon of the gene, two loxP sequences flanking the HBE, a Neo cassette flanked by two FRTs, and a dtA cassette. Transfection was performed by electroporation in two batches of 0.5 ml each in an 0.4 mm gap Biorad cuvette using the Biorad GenePulser and its Capacitance Extender at 200 V and 950 µF capacitance. Selection was performed with 0.2 mg/ml G418. Recombinant clones were further tested by PCR and Southern hybridization.

### Chromatin Immunoprecipitation (ChIP)

ChIP experiments were performed as described [68]. All ChIPs were done in triplicate and analyzed by duplicate qPCRs. Real-time PCR was performed on Roche Lightcycler using Roche SYBR Green mix (Roche, Switzerland). Five genomic regions were chosen on the *Nodal* locus as shown on Figure 6F. The occupancy of these regions was quantified by quantitative PCR analysis of the ratio of the ChIP signal versus the input signal. The following antibodies were used: anti-acetyl K27-Histone H3 (abcam, ab4729) and anti-trimethyl K2-Histone H3 (Millipore, 07-449), and for mock ChIP, anti-GFP (lifetechnologies, A11122). See Materials and Methods S1 for primer sequences.

### Generation of Aggregation Chimeras

*Nodal^condHBE-YFP^* and *Nodal^ΔHBE-YFP^* ES cells were labelled with nuclear mCherry by transfection with a plasmid expressing mCherry under the control of the strong promoter CAG and the neomycin resistance gene. mCherry-positive cells were selected with 0.2 mg/ml G418. Eight-cell stage Swiss × Swiss mouse embryos were collected in M2 (Sigma), shelled in Tyrode's solution (Sigma), and co-cultured in G2 (Vitrolife) at 37°C and 5% CO_2_ with groups of 10–15 of mCherry labelled, *Nodal^condHBE-YFP^*, or *Nodal^ΔHBE-YFP^* ES cells. Aggregated chimeras were cultured in G2 for 60–72 h until they reached the equivalent of stage E4.5 or transferred 36 h later into the uterus (up to 10 blastocysts) of E2.5 pseudopregnant mice, where they developped until they reached the equivalent of stage E6.5.

### Imaging and Image Processing

Acquisitions of fixed embryos were performed at Imago Seine Core Facility using confocal microscopes (Zeiss LSM 710 and 780). See supplementary experimental procedures for details (Materials and Methods S1). The total number of cells and/or of labeled cells was obtained by counting cell nuclei manually. All images shown in the article are one 5 µm confocal section.

## Supporting Information

Figure S1 HBE contains epigenetic signatures characteristic of active enhancers. ChIP-seq data for H3K4me3, H3K27me3, and H3K4me1 were subtracks of the Broad H3 ChIP-seq track in the UCSC genome browser on Mouse Feb. 2006 (NCBI36/mm8) Assembly and represent ChIP-seq density signal. ChIP-seq data for p300 and H3K27ac were wig files corresponding to the reference paper extracted from GEO (Accession GSE24165) and uploaded in the UCSC genome browser (http://genome.ucsc.edu/).
(TIF)

Figure S2 Confirmation of Nanog deletion in RCNβH ES cells and Oct4 inhibition in ZHBTc4 ES cells. (A–B″) RCNβH cells, stained for GFP (A′ and B′) and Nanog (A″ and B″) before (A–A″) and after (B–B″) deletion of *Nanog* by the addition of Tamoxifen. (C–D″) RCNβH cells, stained for GFP (C′ and D′) and Oct4 (C″ and D″) before (C–C″) and after (D–D″) deletion of *Nanog* by the addition of Tamoxifen. (E–F′) ZHBTc4 cells, stained for Oct4 before (E′) and after (F′) inhibition of *Oct4* by the addition of doxycyclin. DAPI stains ESC nuclei. One confocal section. Scale bar, 25 µm.
(TIF)

Figure S3 Pluripotency factor binding sites in HBE. (A) Sequence of HBE. Regions 1–4 are separated by “//”. Subregions a–d within regions 2 and 3 are separated by “/”. Transcription factor binding sites of interest are highlighted. The mutated nucleotides are underlined. Long clusters of transcription factor binding sites that were deleted are in bold characters. Nanog and Oct4 binding sites tested in gel shift assays are in black boxes. (B) Luciferase reporter assays on ESCs using the minimal promoter E1b. Luciferase activity before (HBE23) and after mutation of the main Oct4 binding site (HBE23-O*) or of all three Oct4 binding sites (HBE23-O*). Luciferase activities are shown relative to HBE23 construct fixed to 10 arbitrary units. Bars represent mean ± SD of a minimum of three independent experiments performed for each condition. Ctrl, control E1b vector.
(TIF)

Figure S4 Oct4 specifically binds the identified conserved Oct4 binding site in ESCs. Representative gel-shift assays performed with ES cell extracts and double-strand ^32^P oligonucleotide. (A) ZHBTc4 ES cells (Doxycyclin treated – Z^+^, in which Oct4 was depleted – or not – Z^–^). Oct4 oligonucleotide corresponding to the main Oct4 binding site, WT, or mutated (MUT) as in the luciferase assay constructs (Figure S3B). The migration of WT oligonucleotides were shifted in the presence of Z^–^ cell extract expressing Oct4 (line 5A), but not in absence of Oct4 (Z^+^ cells, line 12A). Oct4 specific antibodies destabilized the complexes (line 6A). This shift was not observed with mutated oligonucleotides (MUT, line 10A). (B) RCNβH ES cells (tamoxifen treated – R^+^, in which Nanog was depleted – or not – R^–^). Nanog oligonucleotide corresponding to the identified Nanog binding site in HBE2a, WT, or mutated (MUT) as in the luciferase assay constructs. The migration of WT oligonucleotides in the presence of R^–^ cell extract expressing Nanog (line 2B) or R^+^ cell extract without any Nanog (6B) was shifted, but not that of mutated oligonucleotides (lines 9B and 11B). This shift was not observed with mutated oligonucleotides (MUT, line 10A). Arrows, nonspecific DNA–protein complexes (not abolished by incubation with the cold probe). Arrowheads, specific DNA–protein complexes. Vertical bar, typical HSF/HSE complexes, loaded as a positive control of the assay to assess the quality of ES cell extracts. HSE (Heat Shock Element) is bound by HSFs, transcription factors highly expressed in ES cells and in preimplantation embryos [69].
(TIF)

Figure S5 Homologous recombination in ESCs. (A) Representation of the homologous recombination strategy. Probes, restriction sites, and the resulting fragments are depicted. (B) Southern blot showing successful targeting of the 5′ end of the homologous recombination construct. 5′ probe used. (C) Southern blot showing successful targeting of the 3′ end of the homologous recombination construct. 3′ probe used. (D) Southern blot showing conservation in the recombinant allele of the 5′ loxP sequence. loxP probe used. (E) Representation of HBE deletion in the recombinant allele. (F) Southern blot showing successful HBE deletion after transfection of the Cre recombinase. Venus probe used. Each gel was photographed after ethidium bromide staining, and the image of the ladder lane was associated with that of the corresponding autoradiogramme.
(TIF)

Figure S6 HBE is dispensable for *Nodal* expression in EpiSCs. (A) Representative RT-qPCR for several different markers confirming the differentiation of ES cells into EpiSCs, in *Nodal* ^condHBE-YFP^(HBE+) and *Nodal* ^ΔHBE-YFP^(HBE–) ES cells during 10 d of differentiation into EpiSCs. (B) Representative RT-qPCR showing changes in *Nodal* and YFP expression of *Nodal* ^condHBE-YFP^(HBE+) ES cells during 10 d of differentiation into EpiSCs. (C–C′) mCherry expression confirming the efficient transfection of the Cre recombinase in *Nodal* ^condHBE-YFP^ EpiSCs cells 6 d after the transfection. The field is the same as in Figure 5D–E. (D) Genomic RT-PCR showing efficiency of conditional HBE allele deletion after transfection with Cre recombinase. (E) RT-PCR showing levels of *YFP* in *Nodal* ^condHBE-YFP^ EpiS cells cultured for 6 d after transfection of Cre recombinase to delete HBE (+Cre).
(TIF)

Materials and Methods S1 Supplementary materials and methods.
(DOCX)

## Abbreviations

AIE/LSE: asymmetric initiatior element/left side specific enhancer
ASE: asymmetric enhancer
ChIP: chromatin immunoprecipitation
DVE: distal visceral endoderm
ESC: embryonic stem cell
EpiSC: epiblast stem cell
FGF: fibroblast growth factor
GRN: gene regulatory network
H3K: histone-3 lysine
HBE: highly bound element
ICM: inner cell mass
MTL: multi-transcription factor-binding loci
NDE: node enhancer
NIS: *Nodal* intervening sequence
ORF: open reading frame
PrE: primitive endoderm
PEE: proximal enhancer element
SBE: Smad binding element
TGF: transforming growth factor
TSS: transcription start site
WT: wild type
YFP: yellow fluorescent protein
