# Supplementary figures and images for: A Novel Nodal Enhancer Dependent on Pluripotency Factors and Smad2/3 Signaling Conditions a Regulatory Switch During Epiblast Maturation
Source: PLoS Biol. 2014 Jun 24;12(6):e1001890. doi: 10.1371/journal.pbio.1001890 (PMC4068991; doi:10.1371/journal.pbio.1001890)

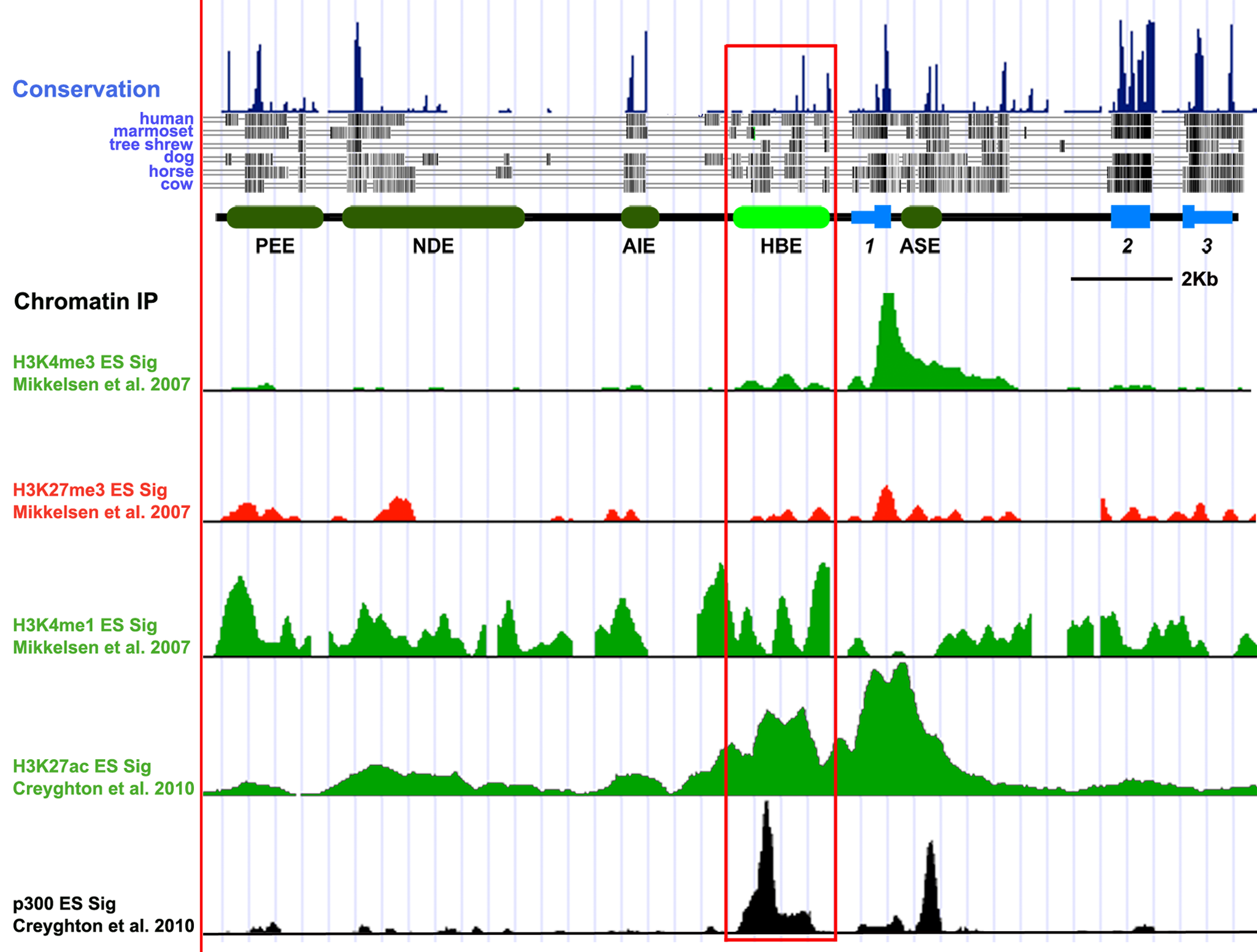

Supplement: Figure S1 — HBE contains epigenetic signatures characteristic of active enhancers. ChIP-seq data for H3K4me3, H3K27me3, and H3K4me1 were subtracks of the Broad H3 ChIP-seq track in the UCSC genome browser on Mouse Feb. 2006 (NCBI36/mm8) Assembly and represent ChIP-seq density signal. ChIP-seq data for p300 and H3K27ac were wig files corresponding to the reference paper extracted from GEO (Accession GSE24165) and uploaded in the UCSC genome browser (http://genome.ucsc.edu/). (TIF) [file pbio.1001890.s001.tif]

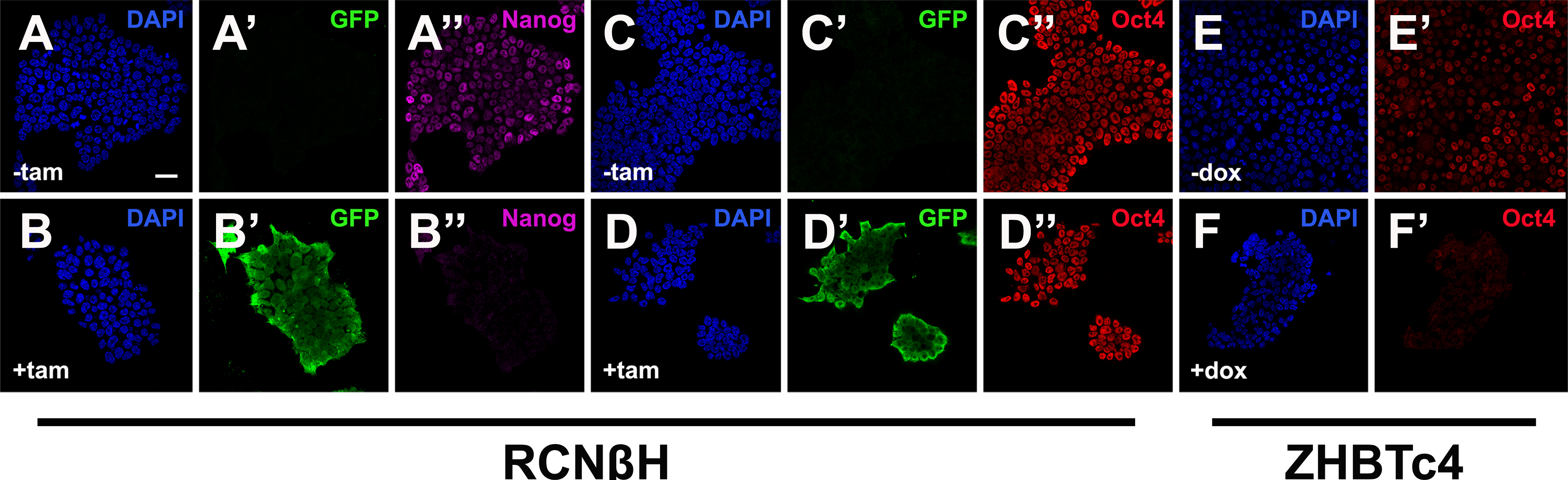

Supplement: Figure S2 — Confirmation of Nanog deletion in RCNβH ES cells and Oct4 inhibition in ZHBTc4 ES cells. (A–B″) RCNβH cells, stained for GFP (A′ and B′) and Nanog (A″ and B″) before (A–A″) and after (B–B″) deletion of Nanog by the addition of Tamoxifen. (C–D″) RCNβH cells, stained for GFP (C′ and D′) and Oct4 (C″ and D″) before (C–C″) and after (D–D″) deletion of Nanog by the addition of Tamoxifen. (E–F′) ZHBTc4 cells, stained for Oct4 before (E′) and after (F′) inhibition of Oct4 by the addition of doxycyclin. DAPI stains ESC nuclei. One confocal section. Scale bar, 25 µm. (TIF) [file pbio.1001890.s002.tif]

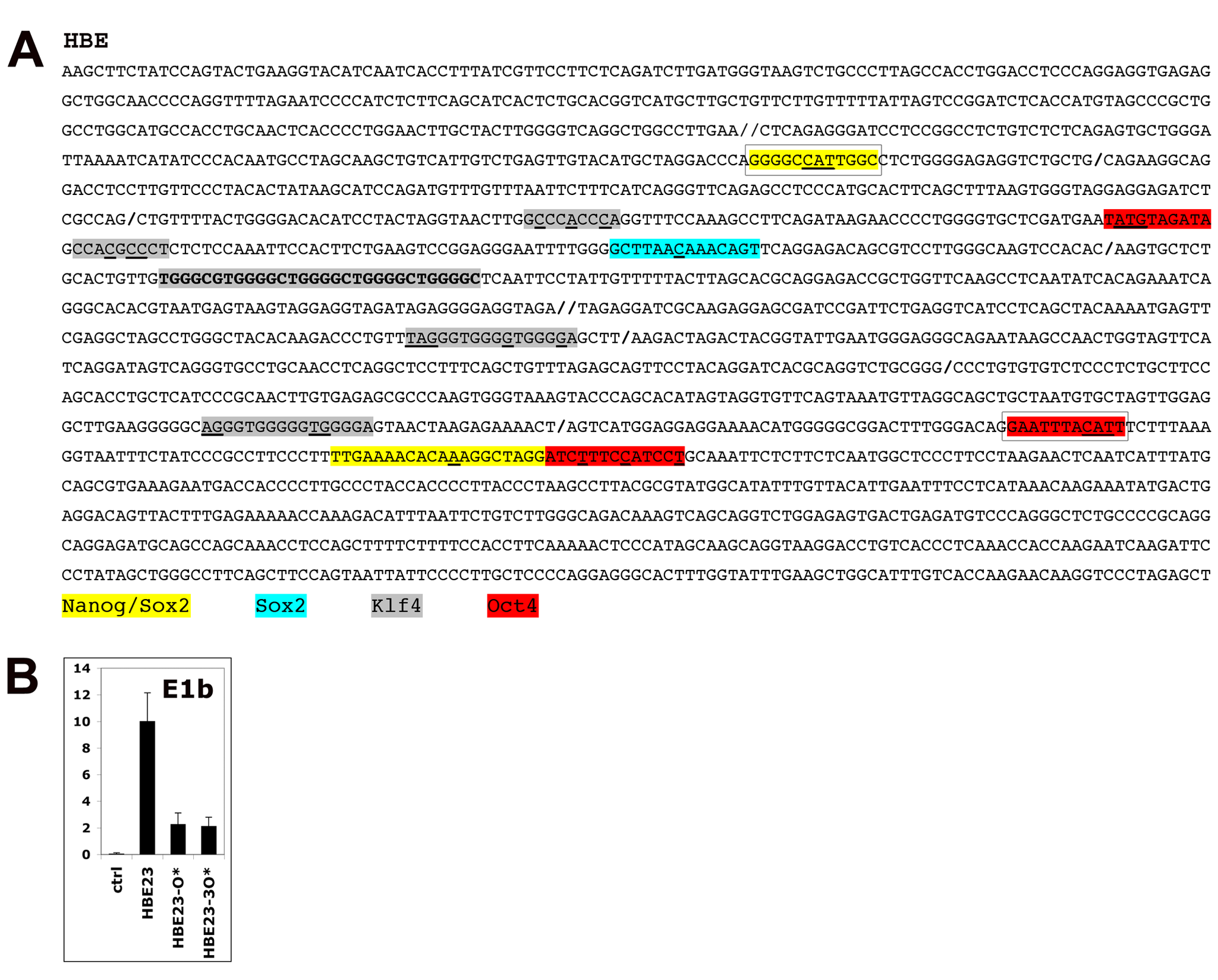

Supplement: Figure S3 — Pluripotency factor binding sites in HBE. (A) Sequence of HBE. Regions 1–4 are separated by “//”. Subregions a–d within regions 2 and 3 are separated by “/”. Transcription factor binding sites of interest are highlighted. The mutated nucleotides are underlined. Long clusters of transcription factor binding sites that were deleted are in bold characters. Nanog and Oct4 binding sites tested in gel shift assays are in black boxes. (B) Luciferase reporter assays on ESCs using the minimal promoter E1b. Luciferase activity before (HBE23) and after mutation of the main Oct4 binding site (HBE23-O*) or of all three Oct4 binding sites (HBE23-O*). Luciferase activities are shown relative to HBE23 construct fixed to 10 arbitrary units. Bars represent mean ± SD of a minimum of three independent experiments performed for each condition. Ctrl, control E1b vector. (TIF) [file pbio.1001890.s003.tif]

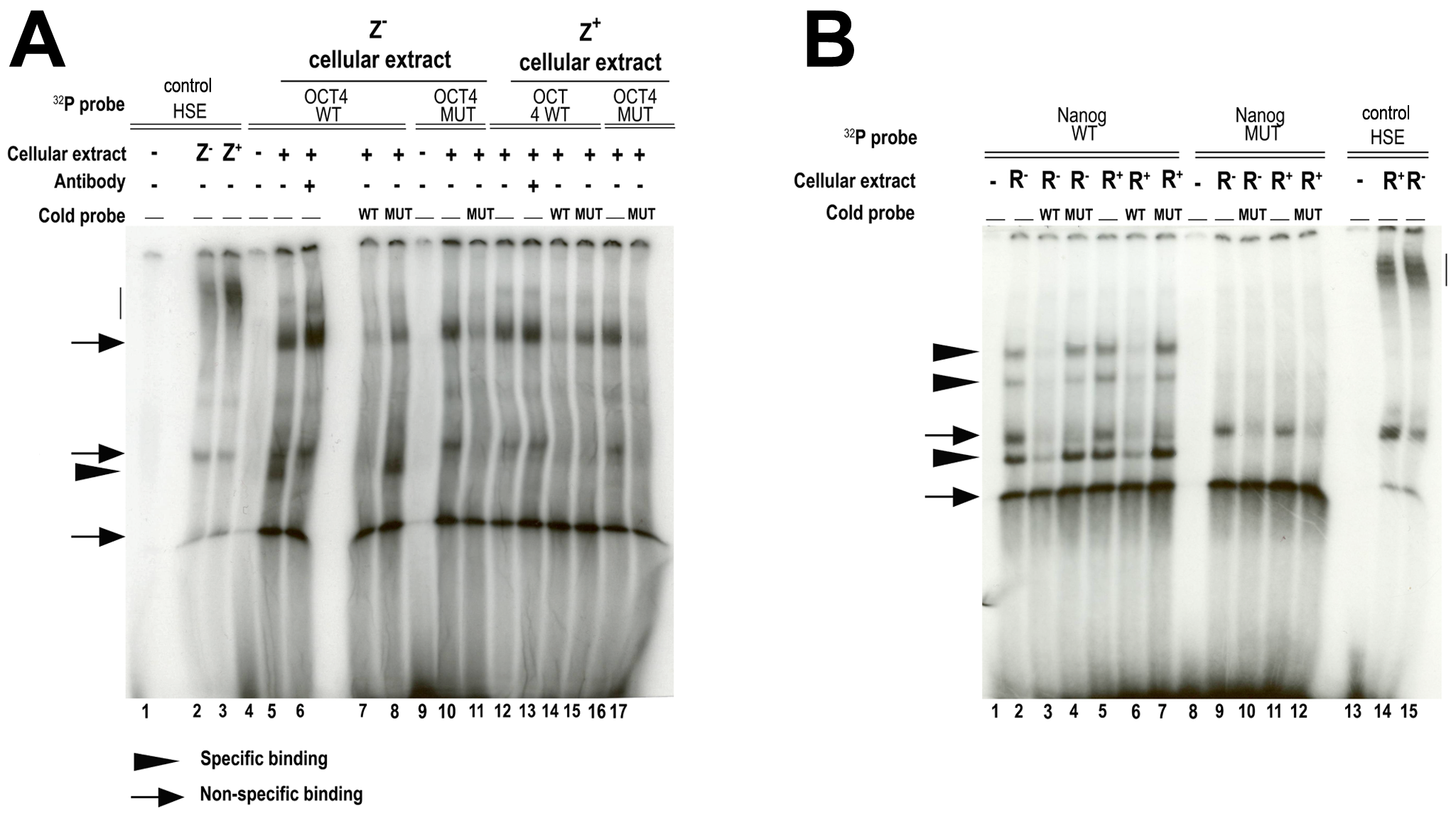

Supplement: Figure S4 — Oct4 specifically binds the identified conserved Oct4 binding site in ESCs. Representative gel-shift assays performed with ES cell extracts and double-strand 32P oligonucleotide. (A) ZHBTc4 ES cells (Doxycyclin treated – Z+, in which Oct4 was depleted – or not – Z–). Oct4 oligonucleotide corresponding to the main Oct4 binding site, WT, or mutated (MUT) as in the luciferase assay constructs (Figure S3B). The migration of WT oligonucleotides were shifted in the presence of Z– cell extract expressing Oct4 (line 5A), but not in absence of Oct4 (Z+ cells, line 12A). Oct4 specific antibodies destabilized the complexes (line 6A). This shift was not observed with mutated oligonucleotides (MUT, line 10A). (B) RCNβH ES cells (tamoxifen treated – R+, in which Nanog was depleted – or not – R–). Nanog oligonucleotide corresponding to the identified Nanog binding site in HBE2a, WT, or mutated (MUT) as in the luciferase assay constructs. The migration of WT oligonucleotides in the presence of R– cell extract expressing Nanog (line 2B) or R+ cell extract without any Nanog (6B) was shifted, but not that of mutated oligonucleotides (lines 9B and 11B). This shift was not observed with mutated oligonucleotides (MUT, line 10A). Arrows, nonspecific DNA–protein complexes (not abolished by incubation with the cold probe). Arrowheads, specific DNA–protein complexes. Vertical bar, typical HSF/HSE complexes, loaded as a positive control of the assay to assess the quality of ES cell extracts. HSE (Heat Shock Element) is bound by HSFs, transcription factors highly expressed in ES cells and in preimplantation embryos [69]. (TIF) [file pbio.1001890.s004.tif]

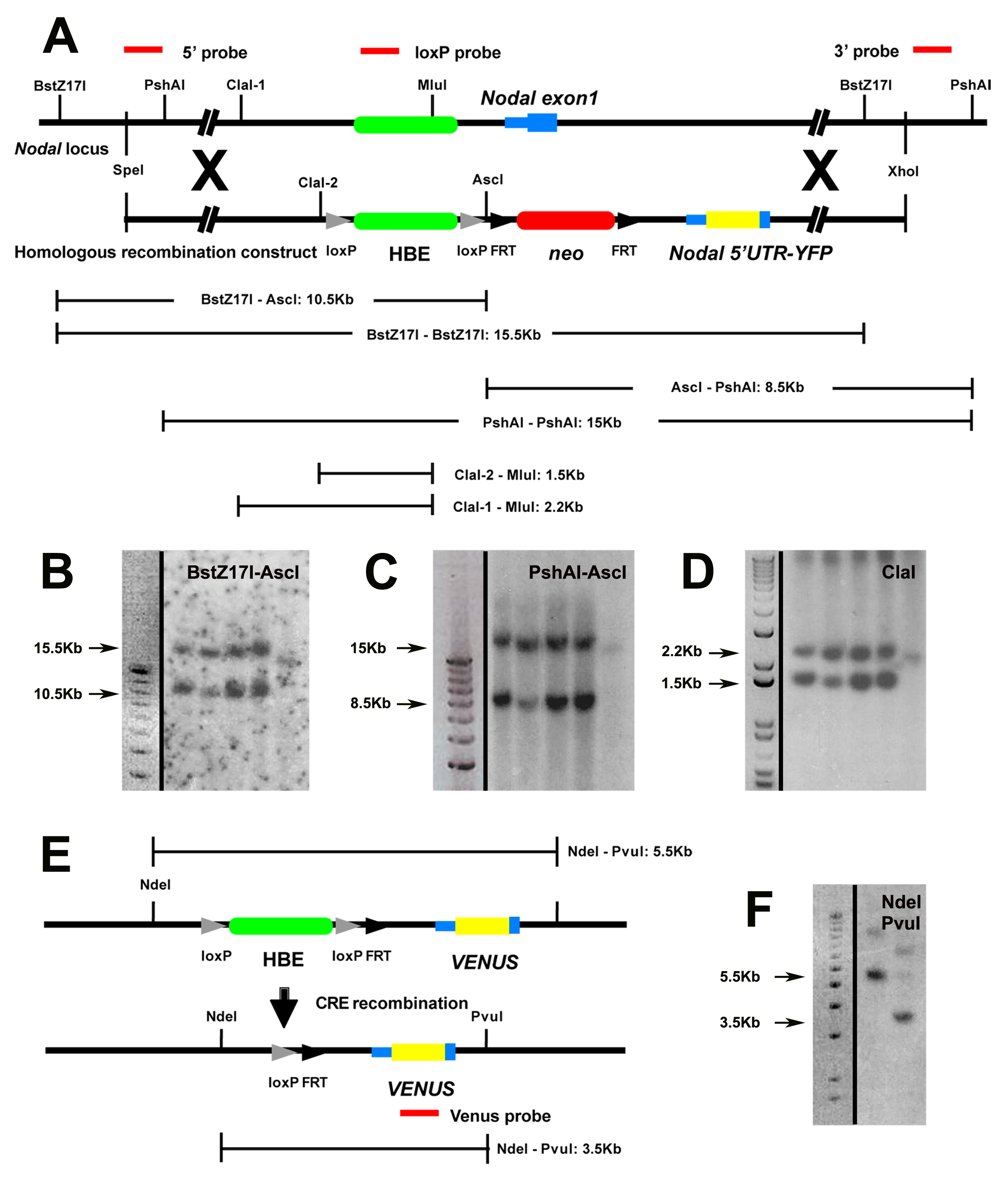

Supplement: Figure S5 — Homologous recombination in ESCs. (A) Representation of the homologous recombination strategy. Probes, restriction sites, and the resulting fragments are depicted. (B) Southern blot showing successful targeting of the 5′ end of the homologous recombination construct. 5′ probe used. (C) Southern blot showing successful targeting of the 3′ end of the homologous recombination construct. 3′ probe used. (D) Southern blot showing conservation in the recombinant allele of the 5′ loxP sequence. loxP probe used. (E) Representation of HBE deletion in the recombinant allele. (F) Southern blot showing successful HBE deletion after transfection of the Cre recombinase. Venus probe used. Each gel was photographed after ethidium bromide staining, and the image of the ladder lane was associated with that of the corresponding autoradiogramme. (TIF) [file pbio.1001890.s005.tif]

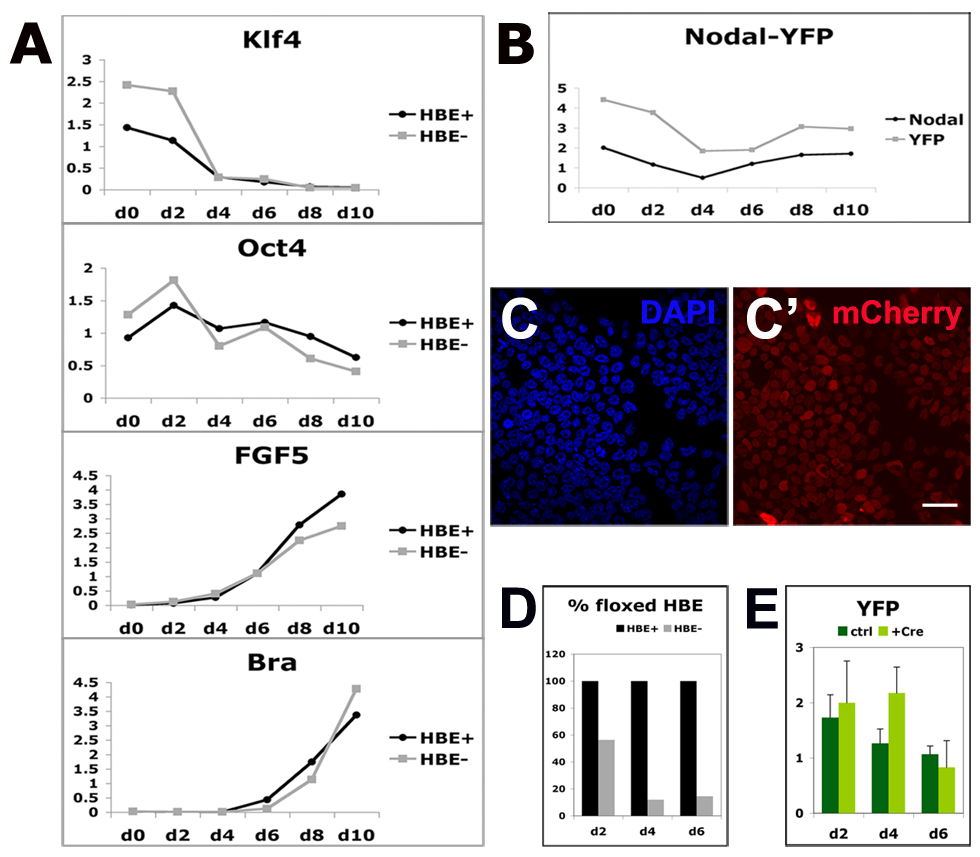

Supplement: Figure S6 — HBE is dispensable for Nodal expression in EpiSCs. (A) Representative RT-qPCR for several different markers confirming the differentiation of ES cells into EpiSCs, in Nodal condHBE-YFP(HBE+) and Nodal ΔHBE-YFP(HBE–) ES cells during 10 d of differentiation into EpiSCs. (B) Representative RT-qPCR showing changes in Nodal and YFP expression of Nodal condHBE-YFP(HBE+) ES cells during 10 d of differentiation into EpiSCs. (C–C′) mCherry expression confirming the efficient transfection of the Cre recombinase in Nodal condHBE-YFP EpiSCs cells 6 d after the transfection. The field is the same as in Figure 5D–E. (D) Genomic RT-PCR showing efficiency of conditional HBE allele deletion after transfection with Cre recombinase. (E) RT-PCR showing levels of YFP in Nodal condHBE-YFP EpiS cells cultured for 6 d after transfection of Cre recombinase to delete HBE (+Cre). (TIF) [file pbio.1001890.s006.tif]
