# Supplementary material for: A Novel Nodal Enhancer Dependent on Pluripotency Factors and Smad2/3 Signaling Conditions a Regulatory Switch During Epiblast Maturation
Source: PLoS Biol. 2014 Jun 24;12(6):e1001890. doi: 10.1371/journal.pbio.1001890 (PMC4068991; doi:10.1371/journal.pbio.1001890)
Supplement: Materials and Methods S1 — Supplementary materials and methods. (DOCX) [file pbio.1001890.s007.docx]

**Supplementary Materials and Methods**

**ES cell culture**

CCE mouse ESCs (a gift by Liz Robertson) and CK35 ESCs (a gift from Chantal Kress) were cultured on mitomycin treated SNL feeders plated on gelatin (Sigma), in Dulbecco’s Modified Eagle’s Medium (DMEM, Gibco) supplemented with 15% heat-inactivated ES-qualified fetal bovine serum (FBS, Gibco) and 0.1 mM β-mercaptoethanol (Sigma) at 37ºC and 5% CO_2_. ZHbTc4 and RCNbH cells (a gift by Veronique Azuara) were cultured on gelatin in Glasgow Minimum Essential Medium (GMEM, Gibco) supplemented with 15% FBS, MEM Non-Essential Amino Acids (MEM-NEAA, Gibco), 1mM Sodium Pyruvate (Gibco), 0.075% Sodium Bicarbonate (Sigma), 0.1mM β-mercaptoethanol, 2mM L-Glutamine (Gibco), 100 U/ml penicillin (Gibco), 0.1mg/ml streptomycin (Gibco) and 1000U/ml LIF (PAA). Cells were passaged every 2 days using 0.05% Trypsin (Gibco)..

**Immunofluorescence**

Antibodies were used in the following combinations: rabbit anti-GFP and donkey anti-rabbit Alexa 488 (both Molecular probes), goat anti-Gata4 (Santa Cruz) or goat anti-Oct4 (Santa Cruz) and donkey anti-goat Alexa 647 (Molecular probes), goat anti-GFP (AbCam) and donkey anti-goat Alexa488 (Molecular probes), rabbit anti-Nanog (Cosmo bio) and donkey anti-rabbit Alexa 568 (Molecular probes). All antibodies were used at a 1/200 dilution except for the anti-Oct4 which was used at a 1/100 dilution. Cortical actin was stained with Alexa 568-conjugated Phalloidin, except when associated with Nanog staining, Alexa 647-conjugated Phalloidin was then used.

**Site directed mutagenesis**

Only the forward sequence is indicated. Lower case designate the mutated bases

Nanog/Sox2-A (5’-GTA CAT GCT AGG ACC CAG GGG C C/t A/g TT/aG GCC TCT GGG GAG AGG TCT G-3’)

Klf4-A (5’-ATC CTA CTA GGT AAC TTG GC/aC CA/tC CC/aA GGT TTC CAA AGC CTT CAG-3’)

Oct4-A (5’- GAT AAG AAC CCC TGG GGT GCT CGA TGA ATA/c T/gG/cT AGA TAG CCA CGC CCT CTC TCC AAA TTC CAC-3’)

Klf4-B (5’-GAT GAA TAT GTA GAT AGC CAC/g GC/tC/a CTC TCT CCA AAT TCC ACT TCT G-3’)

Sox2 (5’-CGG AGG GAA TTT TGG GGC TTA AC/gA AAC AGT TCA GGA GAC AGC GTC C-3’)

Klf4-C (deletion) (5’-CAC AAG TGC TCT GCA CTG TTG TGG CTC AAT TCC TAT TGT TTT TAC TTA GCA CG-3’)

Klf4-D (5’-CTG GGC TAC ACA AGA CCC TGT/g TTA G/tGG TGG GG/tT GGG /tGAG CTT AAG ACT AGA CT-3’)

Klf4-E (5’-AGG CTT GAA GGG GGC A/cG/tG GTG GGG GT/aG/a GGG AGT AAC TAA GAG-3)

Oct4-B (5’-CGG ACT TTG GGA CAG GAA TTT AC/gA/c T/gTT CTT TAA AGG TAA TTT CTA TCC CGC C-3’)

Nanog/Sox2-B (5’-CTA TCC CGC CTT CCC TTT TGA AAA CAC AA/gA GGC TAG GAT CTT TCC ATC CTG CAA ATT CTC-3’)

Oct4-C (5’-CCC TTT TGA AAA CAC AAA GGC TAG GAT CT/aT TCC/g ATC CTG CAA ATT CTC TTC TCA ATG GC-3’)

Additional primers containing both Oct4-A and Klf4-B as well as Nanog/Sox2-C and Oct4-B mutations were used to introduce them together.

**Gel Shift Assay**

Cell extracts were prepared as described [1]. 5.10^6^ cells were submitted to 2–4 rapid freeze-thaw cycles, in 5 volumes of extraction buffer.

Whole cell extracts (40μg of proteins) were incubated with a (^32^P)-labeled oligonucleotide and complexes were separated on a native 4% polyacrylamide gel as described [2]. The components of the retarded complexes were analysed when possible by supershift using antibodies against HSF1, Oct4 or Nanog.

The oligonucleotides used are double strand. The binding sites are underlined, lower case designate the mutated bases:

HSE (5’-CTA GAA CGT TCT AGA AGC TTC GAG A-3’),

Nanog WT (5’- TAG GAC CCA GGG GCC ATT GGC CTC TGG GGA-3’),

Nanog MUT (5’tag gac cca ggg gct gTa ggc ctc tgg gga -3’),

Oct4 WT (5’- TGG GGG CGG ACT TTG GGA CAG GAA TTT ACA TTT CTT TAA AGG TAA TTT CT -3’),

Oct4 MUT (5’- TGG GGG CGG ACT TTG GGA CAG GAA TTT Agc gTT CTT TAA AGG TAA TTT CT -3’).

The components of the retarded complexes were analysed when possible by supershift using antibodies against HSF1 (10ng/μl final; 3E2 Neomarkers), Oct4 (70ng/μl final; SC8628 SantaCruz).

**Imaging and image processing**

For E2.5–E5.5 embryos a 40x oil Plan-Apochomat  NA 1,3 objective (Zeiss) was used. E6.5-E7.5 embryos were acquired using a 25x oil Plan-Apochomat  NA 0,8 objective (Zeiss). E8.5 embryos were acquired using 10x objective. The acquisition software used was ZEN (Zeiss). Image stacks were performed using ImageJ software (<http://rsbweb.nih.gov/ij/>).

**RT-PCR primers:**

Venus Forward: (5’- AAG GAC GAC GGC AAC TAC AA -3’)

Venus Reverse: (5’- AAG TCG ATG CCC TTC AGC TC -3’)

Nodal Forward: (5’- CTG TGA GGG CGA GTG TCC TA -3’)

Nodal Reverse: (5’- CAG TGG CTT GGT CTT CAC GG -3’)

GAPDH Forward: (5’- TTC AAC AGC AAC TCC CAC TCT TC -3’)

GAPDH Reverse: (5’- CCC TGT TGC TGT AGC CGT ATT C -3’)

POU5f1 (oct4) Forward: (5’- AGT TGG CGT GGA GAC TTT GC -3’)

POU5f1 Reverse: (5’- CAG GGC TTT CAT GTC CTG G -3’)

KLF4 Forward: (5’- GGA AGG GAG AAG ACA CTG CG -3’)

KLF4 Reverse: (5’- ATG TGA GAG AGT TCC TCA CGC C -3’)

FGF5 Forward: (5’- TGT ACT GCA GAG TGG GCA TC -3’)

FGF5 Reverse: (5’- ACA ATC CCC TGA GAC ACA GC -3’)

Brachyury Forward: (5’- CCA GCT CTA AGG AAC CAC CG -3’)

Brachyury Reverse: (5’- ACT CCG AGG CTA GAC CAG TT -3’)

**ChIP Primers :**

Nodal Region 1 Forward: (5’- GGGGAAGCACCATGAGTTGA -3’)

Nodal Region 1 Reverse: (5’- CCCATGTCCAAGACACCTGA -3’)

Nodal Region 2 Forward: (5’- GGATGAACTGGAGAGGACCG -3’)

Nodal Region 2 Reverse: (5’- CTACCTCGGGAAGTCTCCCT -3’)

Nodal Region3 Forward: (5’- CCTGTTGGGCTCTACTCCAC-3’)

Nodal Region 3 Reverse: (5’- GAGGCTCAACATGTACGCCA -3’)

Nodal Region 4 Forward: (5’- GCCCATGTCTTGTGCCCAG -3’)

Nodal Region 4 Reverse: (5’- CATGTACGCCAGAGGGGAC -3’)

Nodal Region 5 Forward: (5’- CCCAGCGACCTGAGTGATG -3’)

Nodal Region 5 Reverse: (5’- CCCAAGAGGCGAGATGTTGA -3’)

References

1. Mezger V, Bensaude O, Morange M (1989) Unusual levels of heat shock element-binding activity in embryonal carcinoma cells. Mol Cell Biol 9: 3888-3896.

2. Rallu M, Loones M, Lallemand Y, Morimoto R, Morange M, et al. (1997) Function and regulation of heat shock factor 2 during mouse embryogenesis. Proc Natl Acad Sci U S A 94: 2392-2397.
